# *Caliciviridae* Other Than Noroviruses

**DOI:** 10.3390/v11030286

**Published:** 2019-03-21

**Authors:** Ulrich Desselberger

**Affiliations:** Department of Medicine, University of Cambridge, Addenbrooke’s Hospital, Cambridge CB2 0QQ, UK; ud207@medschl.cam.ac.uk

**Keywords:** Bavovirus, Lagovirus, Nebovirus, Norovirus, Recovirus, Sapovirus, Valovirus, Vesivirus, enteroids, reverse genetics, animal models

## Abstract

Besides noroviruses, the *Caliciviridae* family comprises four other accepted genera: *Sapovirus, Lagovirus, Vesivirus*, and *Nebovirus*. There are six new genera proposed: *Recovirus, Valovirus, Bavovirus, Nacovirus, Minovirus*, and *Salovirus.* All *Caliciviridae* have closely related genome structures, but are genetically and antigenically highly diverse and infect a wide range of mammalian host species including humans. Recombination in nature is not infrequent for most of the *Caliciviridae*, contributing to their diversity. Sapovirus infections cause diarrhoea in pigs, humans and other mammalian hosts. Lagovirus infections cause systemic haemorrhagic disease in rabbits and hares, and vesivirus infections lead to lung disease in cats, vesicular disease in swine, and exanthema and diseases of the reproductive system in large sea mammals. Neboviruses are an enteric pathogen of cattle, differing from bovine norovirus. At present, only a few selected caliciviruses can be propagated in cell culture (permanent cell lines or enteroids), and for most of the cultivatable caliciviruses helper virus-free, plasmid only-based reverse genetics systems have been established. The replication cycles of the caliciviruses are similar as far as they have been explored: viruses interact with a multitude of cell surface attachment factors (glycans) and co-receptors (proteins) for adsorption and penetration, use cellular membranes for the formation of replication complexes and have developed mechanisms to circumvent innate immune responses. Vaccines have been developed against lagoviruses and vesiviruses, and are under development against human noroviruses.

## 1. Introduction

Noroviruses (NoVs) are a major cause of epidemic acute gastroenteritis (AGE), and most chapters of this Special Issue are devoted to the structure, replication, pathogenesis, immune responses, epidemiology/evolution, and vaccine development relating to viruses of this genus of the *Caliciviridae* family. Murine norovirus (MuNoV) has received particular attention, since it can be propagated in cell culture [1], can be manipulated in well-developed reverse genetics systems [2,3,4,5] and has become a potent animal model of molecular-biological research [6,7,8,9]. Human noroviruses (HuNoVs) can now also be propagated in human B cells in the presence of histo-blood group antigen (HBGA) expressing bacteria or of synthetic H-type HGBA [10] or in human enteroids [11]. Reverse genetics systems for HuNoVs are under development, whereas an animal model for HuNoVs infection is in its infancy [12]. Vaccine development against human NoV disease is under intense study [13]. The present chapter aims at providing an overview on genera of the *Caliciviridae* family other than noroviruses*.

*The review on *Caliciviridae* other than noroviruses complements the many contributions to NoV research in this Special Issue of *Viruses* which relate to:

- NoV structure [14];
- The molecular biology of NoV replication [15];
- NoV replication in the immunocompromised host [16];
- Factors affecting host susceptibility to NoV infection [17];
- In vitro propagation of NoVs [18];
- Innate and acquired immune responses to NoV infections [19];
- Molecular epidemiology of HuNoVs [20];
- Use of animal models of NoV infection [21];
- Treatment and development of antivirals [22];
- Vaccine development [23].

## 2. Classification

The *Caliciviridae* family contains five genera: *Norovirus, Sapovirus, Lagovirus, Vesivirus*, and *Nebovirus.* Their taxonomy including prominent virus species is presented in Table 1. At least six additional genera, *Recovirus, Valovirus, Bavovirus, Nacovirus, Minovirus and Salovirus*, have been proposed (Table 1), and more may be discovered by metagenomic analyses.

## 3. Genome Structure

**Figure 1:**
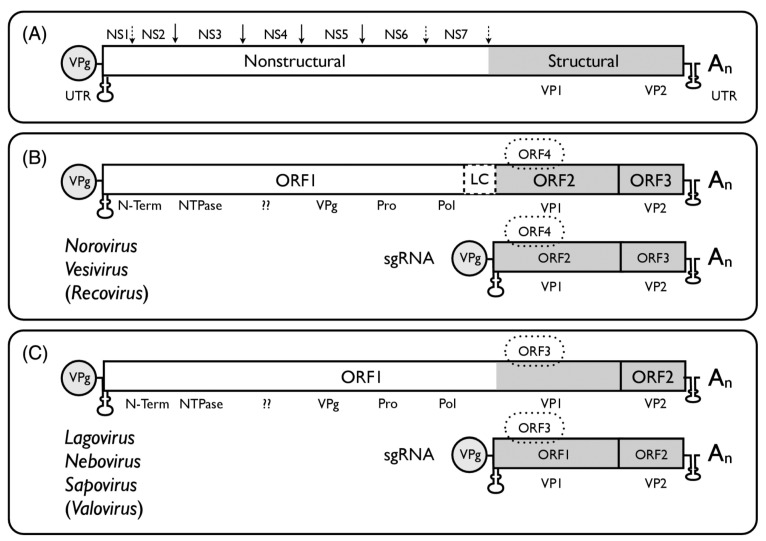
Schematic genome organization of viruses in different genera of the *Caliciviridae*. (**A**) The long ORF encodes a polyprotein consisting of seven mostly non-structural proteins (ORF1) and 2–3 structural proteins (ORF2–ORF4). (**B**) NS3 encodes an NTPase, NS5 the VPg (a structural protein), NS6 a protease, NS7 the RNA-dependent RNA polymerase (RdRp). NS1, NS2 (NS1 + NS2 are also called N-Term), and NS4 have functions in the formation of membranes of the viral replication sites and interact with proteins of the endoplasmic reticulum [32,33]. (**C**) For *Norovirus* and *Vesivirus* ORF2 encodes the major structural protein VP1 (for *Vesivirus* including a precursor N-terminal leader protein (LC). For *Sapovirus, Lagovirus*, and *Nebovirus* the VP1 is derived from the C-terminus of ORF1. ORF2 or ORF3 encode VP2, and ORF4 encodes a protein which has been identified as virulence factor 1 (VF1) for MuNoV. The *Nacovirus* [28,29], *Minovirus* [31], and *Salovirus* [30] genomes have structures as shown in panel C for *Lagovirus, Nebovirus, Sapovirus*, and *Valovirus*. From references [24,28,29,30,31], with permission of authors and publisher.

The genome organisation of the different calicivirus genera (Figure 1, reference [24]) follows the general scheme of a single-stranded, positive-sense RNA of 7.3–8.5 kb in size with a VPg covalently linked to the 5’ end and a long open reading frame (ORF) encoding a polyprotein positioned between relatively short 5’ and 3’ untranslated regions (UTR). By genome structure, the *Caliciviridae* can be differentiated further into two main groups. In the *Norovirus*, *Vesivirus*, and *Recovirus* genera ORF1 is separated from ORF2 and ORF3 near the 3’ end, and an ORF4 (comprised within ORF2) was found in MuNoV, encoding a virulence factor, VF1. The other genera (*Sapovirus*, *Lagovirus*, *Nebovirus*, *Valovirus*) possess a large ORF1 and a standard ORF2 (equivalent to ORF3 of norovirus); ORF3 (equivalent to MuNoV ORF4) has been proposed for sapovirus.

## 4. Replication

The replication cycle for MuNoVs has been studied in most detail [24,34]. Binding of virus to cellular attachment factors (glycans) and additional interaction with co-receptors (proteins) are followed by viral entry into the cell and release of the viral genome into the cytoplasm where it is translated. While cellular mRNAs are capped, NoV RNAs use the viral protein (VPg) which is covalently linked to the 5’ ends of both full-length genomic (g) and subgenomic (sg) RNAs, for the recruitment of cellular translation factors and ribosomes. The viral polyprotein translated from gRNA is processed by successive cleavages. Viral proteins and RNA form membrane-bound replication complexes where viral RNA replication occurs via a double-stranded RNA intermediates and where VPg-linked gRNAs and sgRNAs are produced. VP1 and VP2 are expressed from sgRNAs. Only the gRNA is then encapsidated, and particles are released by lytic or non-lytic mechanisms. The replication cycles of other members of the *Caliciviridae* are similar, as far as they have been studied.

Norovirus recombination appears to be a not infrequent event in nature, although it is difficult to reproduce in the laboratory [35,36].

### 4.1. Sapovirus

**Figure 2:**
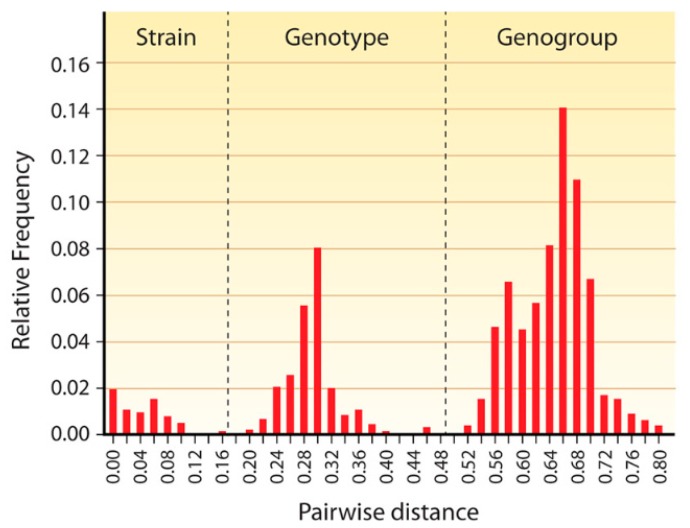
Pairwise distance distribution histogram of complete VP1 genes of 59 sapoviruses. The peaks of 0–0.159, 0.198–0.471, and 0.522–0.807 correspond to the distance range of strains, genotypes, and genogroups, respectively. The cut-off values for genotype and genogroup clusters were <0.169 and <0.488, respectively, and are indicated by vertical dashed lines. From reference [37], with permission of authors and publisher.

Besides NoVs, sapoviruses (SaVs) are members of the only other *Calicivirus* genus able to infect humans of all ages and to cause AGE, which can occur sporadically or in outbreaks. SaVs also infect pigs, mink, dogs, sea lions, and bats [37]. Like NoVs, SaVs are genomically highly diverse; so far 15 genogroups (GG) based on VP1 gene sequences have been distinguished [37,38], four of which (GGI, GGII, GGIV, and GGV) infect humans [39]. Pairwise genomic distance comparison has yielded three clearly distinct, non-overlapping peaks which represent strains, genotypes and genogroups [37] (Figure 2). (Very similar data have been obtained by pairwise genomic distance comparisons for members of many other virus families.) Within genogroups, various genotypes (GT) have been differentiated [37]. Of all genes, those encoding RdRp are most conserved. The ORFs encoding the RdRp and VP1 overlap in the so-called “junction region” which is also a hotspot for recombination [40,41]. Metagenomic identification of SaV sequences has enlarged the diversity of this genus [42]. Highly divergent sapovirus-like calicivirus genome sequences have been identified metagenomically in bats [43,44], which may play an important role in the spreading of SaVs [45].

The porcine SaV (Cowden strain) can be propagated in LLC-PK cells in the presence of intestinal content or bile acids [46], attempts to cultivate human SaVs in cell culture have so far been unsuccessful [47]. The replication cycle of the porcine SaV has been analysed in some detail. Sialic acid residues in α2.3- and α2.6-linkage act as attachment receptors for porcine SaV [48]. Porcine SaV enters cells via clathrin- and cholesterol-dependent endocytosis requiring the presence of dynamin [49]. Occludin acts as a co-receptor [50]. The cellular cap-binding protein eIF4E and RNA helicase eIF4A associate with VPg and are required for SaV translation initiation [51,52]. The PI3K/Akt and MEK/ERK signalling pathways are activated during viral entry [53]. The cyclooxygenase/prostaglandin E2 pathway is involved in SaV replication, and its blockage by specific siRNAs interferes with SaV replication [54]. A helper virus-free reverse genetics system for porcine SaV has been established [55].

SaVs of different genogroups and genotypes co-evolve in humans, preferential infection with particular genotypes, such as with HuNoV GII.4, has not been observed [37]. The overall prevalence of SaVs in low- and middle-income countries is 6.5% [56] and in high-income countries 2.2–12.7% [37].

Recently, a nucleoside acting as a viral polymerase inhibitor has been identified, which blocks the transcription of MuNoV, rotavirus, and SaV genomes in cell culture and—for rotavirus—acts as an effective antiviral in an adult mouse model [57].

### 4.2. Lagovirus

The genus *Lagovirus* contains two main species, *Rabbit hemorrhagic disease virus* (RHDV) and *European brown hare syndrome virus* (EBHSV). Due to a relatively close genomic relationship of RHDV and EBHSV, it has recently been proposed to subsume them under one species, subdivided into two genogroups (GGI, GGII) and several genotypes within genogroups [58]. Lagoviruses can cross-infect within lagomorph host species, and recombination events following dual infections are frequent [59,60,61,62,63]. Lagoviruses use host cell histo-blood group antigens (HBGA) A, B, and H (GGI) or terminal N-acetylglucosamine (GGII) as primary attachment factors [64]. After engagement with the cellular receptor, B cell epitopes on VP60, the major capsid protein, and their binding to HBGA H2 have been defined by monoclonal antibodies [65]; the binding can be blocked by immune sera of vaccinated animals [66]. Following attachment, RHDV is internalized by interaction of rabbit nucleolin with N-terminal residues of VP60 [67], and internalization can be blocked by epitope-specific peptides [67]. The viral genome-linked protein (VPg) was found to be essential for translation initiation [68]. RHDV can be propagated *in vitro* [69]. A reverse genetics system for *Lagovirus* has been described [70].

RHDV grows in multiple organs of infected animals, mainly in the liver, but also in the spleen and blood mononuclear cells, and causes disseminated intravascular coagulation, leading to multiple organ haemorrhages and necroses [71,72]. RHDV RNA has been detected in several wild small mammalian species [73]; however, no RHDV replication has been detected in mice, not even in immunodeficient animals [74]. Thus, lagovirus replication is considered to be restricted to lagomorphs as host species. Rabbits are an animal model for RHDV infection [75]. Particular RHDV strains have been demonstrated to spread rapidly within whole continents such as Australia [76]. Disease control in rabbit breeding facilities relies on biosecurity measures and vaccination [71]. Effective vaccines against RHDV infection have been produced [77,78,79,80]. On the other hand, the virus has been used with favourable outcome as biocontrol against rabbits in Australia, helped by the fact that the virus was not transmitted to non-target host species [81]. RHDV-related non-pathogenic rabbit calicivirus (RCV) strains are endemic in New Zealand and Australia, and there is evidence that prior exposure of rabbits to RCV reduces the frequency of and mortality from RHDV infection [82,83].

### 4.3. Vesivirus

*Feline calicivirus* (FCV) is the major representative of this *Calicivirus* genus, causing mainly upper and rarely lower respiratory tract diseases. Some virulent strains cause systemic disease, including subcutaneous oedema, necroses in multiple organs (liver, spleen, pancreas) and interstitial pneumonia [84,85]. Until recently, it was the first and only calicivirus which could be propagated in various cell lines, such as Vero cells, BHK cells, 293 cells [86], and also in feline mammary epithelial cells [87]. Other vesiviruses are *Vesicular exanthema of swine virus*, which causes vesicular disease and foetal damage in swine [88,89], and the *San Miguel sea lion virus*, which produces exanthemas and diseases of the reproductive system in large sea mammals [90,91].

FCV enters cells by first binding to α2,6-linked sialic acid as an attachment receptor and then interacts with the feline junctional adhesion molecular A (fJAM-A), which serves as a functional co-receptor [86,92]. Recently it has been demonstrated that upon the interaction of FCV particles with a soluble fJAM A fragment, 12 molecules of the minor capsid protein VP2 form large portal entry-like assemblies at a three-fold axis of symmetry, with the hydrophobic N-termini of VP2 pointing away from the viral surface. This leads to the opening of a pore in the capsid shell through which the viral genome may be delivered into the cytoplasm of a host cell [93]. Since all caliciviruses encode a VP2, these morphological changes upon viral entry may be a general phenomenon [93].

Due to its longstanding cultivability, there is more detailed information on the FCV replication cycle. Conserved amino acid residues in the capsid protein of FCV were identified which were essential for interaction with fJAM-A [94]. Knockdown of nucleolin (NCL), a phosphoprotein involved in ribosome biogenesis, was shown to reduce FCV protein synthesis and the yield of viral progeny; but the binding of NCL to both ends of the FCV RNA stimulated its translation [95]. Inactivation of NCL by the aptamer AGRO100 could be reversed by NCL overexpression [95]. Infection of cells with FCV led to activation of the COX-2 PGE2 pathway, which promotes viral replication [96]. siRNA-induced inhibition of COX-2 blocked viral replication, but this could be restored by the addition of PGE2 [96]. One of the non-structural proteins of FCV, p39, was shown to suppress IFN type I production by preventing IRF-3 activation [97]. FCV-infected cells did not produce stress granules (SG), since the viral protease (encoded by ORF NS6) cleaved one of the SG-activating proteins [98]. Host gene expression at the transcriptional level was prevented (“shut off”) by FCV-encoded protease-polymerase protein [99]. Several plasmid only-based reverse genetics systems were developed for FCV [100,101]. Although FCV genomes were found to vary extensively in a European surveillance study, antibodies raised against an FCV isolate obtained more than five decades ago broadly cross-reacted with and cross-neutralized contemporary FCV strains [102]. Cats are a natural model for FCV infection [103]. FCV vaccines were developed more than 10 years ago [104]. Recent FCV vaccine-induced antibodies were found to be of considerable cross-NT activity, in particular when dual vaccine preparations were used [105]. Intranasal immunization with inactivated FCV particles was found to be particularly effective in conveying robust protection [106].

Vesiviruses have also been recognized as infectious agents for household dogs in Italy [107] and more recently in association with an outbreak of acute haemorrhagic gastroenteritis in dogs in the USA [108]. The canine caliciviruses (CCaV) were found to be related to so-called 2117-like vesiviruses originally discovered as contaminants of Chinese hamster ovary cells [109]. Since CCaVs cross-react with the sera of cats, cross-infection of dogs with FCV or possibly infection of cats with 2117-like viruses were suggested [109]. Vesivirus 2117 is able to infect various cell lines [110]. Interestingly, morphologically the capsids of 2117-like vesiviruses more closely resemble sapovirus- and lagovirus-like particles than those of other vesiviruses [111].

The Hom-1 calicivirus, isolated from a human laboratory worker in 1998 as an inadvertent transmission of the San Miguel sea lion virus [90], was found to use the human JAM-1 (hJAM-1) molecule as its receptor in HepG2, HuH7 and SK-CO15 cells [112].

Some authors have suggested that vesivirus infection in humans may be quite common and possibly be associated with hepatitis of unknown but suspected infectious cause [113,114].

### 4.4. Nebovirus

In 2006 the sequence of a cattle-pathogenic enteric virus, Newbury agent-1, was determined and proposed as a new genus, *Nebovirus*, of the *Caliciviridae* [115]. Nebovirus interacts with a wide spectrum of HBGAs [116]. Recombination in different regions of the nebovirus genome was observed [117,118], similar to that in other genera of the *Caliciviridae*. Infection of calves with bovine NoV and nebovirus appears to be common in the USA [119], Brazil [120], Turkey [121], Iran [122], Korea [123], and China [124,125].

### 4.5. Recovirus

A rhesus macaque was the source of a novel calicivirus, named Tulane Virus (TV), which although most closely related to NoVs, was sufficiently different in nucleotide sequence from other caliciviruses to justify classification in a new genus, *Recovirus* [126]. Recoviruses have considerable genomic and antigenic diversity [127], and different genogroups and genotypes can be distinguished, similar to the HuNoV classification [127]. Tulane virus grows in monkey kidney cells and relies on HBGAs as a primary attachment factor [128,129,130]. A reverse genetics system for Tulane virus has been established [131]. Biologically, Tulane virus behaves similar to human NoVs, such as being shed in feces in large quantities and being transmitted by the fecal-oral route, and it has been proposed as a surrogate model for human NoV AGE [132]. Recovirus infection elicits RIG-I- and MDA-5-dependent innate immune responses at different stages of recovirus replication [133]. Recovirus-like viruses were found in the feces of Amerindian children in isolated villages of the Amazonian region of Brazil [134], and recovirus-specific neutralizing antibodies were observed in zookeepers who were in contact with monkeys [135]. A recovirus-related virus, which however had human NoV-like capsid sequences, was recently isolated from bats [136].

### 4.6. Valovirus

**Figure 3:**
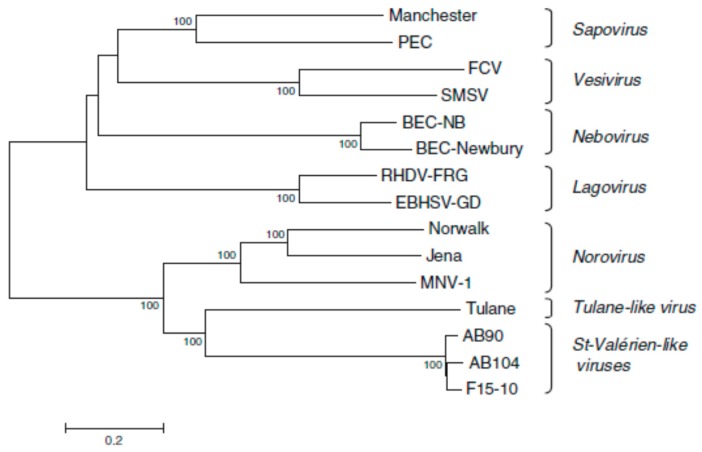
Phylogenetic tree based on nucleotide sequences of complete calicivirus genomes differentiating *Valovirus* as a separate genus. The tree was constructed by the neighbour-joining method MEGA 3.1. The confidence values at the branch points are based on 1000 bootstrap analyses. The calibration bar indicates distance expressed as nt substitutions per site. Abbreviations: BEC-NB, bovine enteric calicivirus NB/80/US; BEC-Newbury, bovine enteric calicivirus Newbury; EBHSV-GD, European brown hare syndrome virus GD strain; FCV, feline calicivirus; Jena, bovine enteric norovirus strain Jena; Manchester, human sapovirus Manchester; MNV-1, mouse norovirus 1; Norwalk, Norwalk virus; PEC, porcine enteric calicivirus; RHDV-FRG, rabbit haemorrhagic disease virus Germany; SMSV, San Miguel Sea Lion Virus; Tulane, Tulane virus; and St Valérien, St Valérien strains AB90, AB104, F15-10. From reference [25], with permission of authors and publisher.

Between 2005 and 2007, caliciviruses isolated from pigs in Canada were proposed as a new genus, *Valovirus* [25] (Figure 3). Similar viruses were detected in pigs in Italy in 2010 [26,27] and more recently isolated from bats in Hungary [43].

### 4.7. Bavovirus and Nacovirus

**Figure 4:**
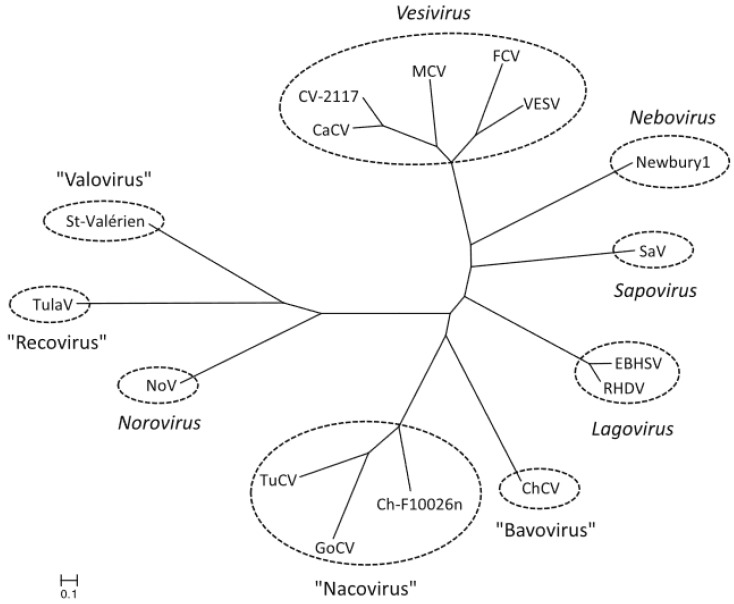
Phylogenetic relationship between the *Nacovirus* genus and other genera of the *Caliciviridae*, based on full length amino acid sequences of VP1, the major capsid protein. The calibration bar indicates genetic distance. Abbreviations: CaCv, canine calicivirus; ChCV, chicken calicivirus Bavaria; Ch-F10026n, chicken calicivirus F10026n; CV-2117, calicivirus 2117; EBHSV, European brown hare syndrome virus; FCV, feline calicivirus; GoCV, goose calicivirus; MCV, mink calicivirus; Newbury1, Newbury-1 virus; NoV, norovirus; RHDV, rabbit haemorrhagic disease virus; SaV, sapovirus; St Valérien, St Valérien virus; TuCV, turkey calicivirus; TulaV, Tulane virus; VESV, vesicular exanthema of swine virus. From reference [29], with permission of authors and publisher.

Between 2009 and 2011 caliciviruses were isolated from chicken and turkey from German farms. Phylogenetic analysis of partial deduced amino acid sequences of the non-structural protease and polymerase genes (320–350 aa) and of the VP1 gene (530–700 aa) strongly suggested the presence of new genera, and the names *Bavovirus* (chicken) and *Nacovirus* (turkey) were proposed [28]. Of 24 chicken farms tested, 83% were positive for bavovirus, 46% were positive for nacovirus, and 38% were positive for both [28]. A naco-like virus was also isolated from a goose [29] (Figure 4).

### 4.8. Salovirus and Minovirus

Novel caliciviruses have been isolated from fish and have been proposed as new genera: *Salovirus* (from Atlantic salmon) and *Minovirus* (from bitterfish) [30,31].

## 5. Concluding Remarks

Major progress in calicivirus research has been made by the availability of a combination of tools:

- The ability to propagate viruses in cell/enteroid culture;
- The availability of helper virus-free (plasmid only-based) reverse genetics systems;
- The availability of suitable animal models.

These tools have been achieved for MuNoV and are close to being fulfilled for HuNoVs. The degree to which these pre-conditions have been accomplished for the other genera of the *Caliciviridae* is summarized in Table 2. Progress is considered to be very advanced for the genera *Lagovirus* and *Vesivirus*, with the other genera requiring further investigation. Recombination in nature has been documented for *Sapovirus, Lagovirus*, and *Nebovirus*, and thus seems to be a general feature of the caliciviruses. Since many genera of the *Caliciviridae* contain important human and animal pathogens, future research will be driven by scientific curiosity as well as public health and economic needs.

## Figures and Tables

**Table 1:** Calicivirus classification including genera and type species (and abbreviations used).

Genus	Type Species
Established:	
*Norovirus* (NoV)	*Norwalk virus* (NV)
*Sapovirus* (SaV)	*Sapporo virus* (SV)
*Lagovirus* (LaV)	*Rabbit hemorrhagic disease virus* (RHDV)
	*European brown hare syndrome virus* (EBHSV)
*Vesivirus* (VeV)	*Vesicular exanthema of swine virus* (VESV)
	*Feline calicivirus* (FCV)
	*San Miguel sea lion virus* (SMSV)
*Nebovirus* (NeV)	*Newbury-1 virus* (NBV)
Proposed:	
*Recovirus* (ReV)	Tulane virus (simian)
*Valovirus* (VaV)	St Valérian virus (porcine)
*Bavovirus* (BaV)	Bayern virus (avian)
*Nacovirus* (NaV)	*Novel avian calicivirus* (chicken, turkey)
*Salovirus* (SaV)	*Atlantic salmon calicivirus* (salmon)
*Minovirus* (MiV)	*Fathead minnow calicivirus* (minnow)

Modified from: http://ictvonline.org/virusTaxonomy.asp 2018 and references [24,25,26,27,28,29,30,31].

**Table 2:** Tools available for comprehensive study of different genera of the *Caliciviridae*.

Genus	Cell/Enteroid Culture	Helper Virus-Free RG System	Animal Model
*Norovirus*			
MuNoV	+ [1]	+ [2,3,4,5]	+ [6,7,8,9]
HuNoV	+ [10,11]	(+) to be published	(+) [12]
*Sapovirus*	+ porcine [46]	+ [55]	+ [37]
	- human [47]		
*Lagovirus*	+ [69]	+ [70]	+ [75]
*Vesivirus*	+ [86,87,110]	+ [100,101]	+ [103]
*Nebovirus*	To be done	To be done	To be done
*Recovirus*	+ [128]	+ [131]	To be done

The genera *Valovirus, Bavovirus, Nacovirus, Minovirus*, and *Salovirus* have only recently been identified, and the techniques mentioned above have to be developed.
